# Evolution of Abscisic Acid Signaling Module and Its Perception

**DOI:** 10.3389/fpls.2020.00934

**Published:** 2020-07-10

**Authors:** Yufei Sun, Oded Pri-Tal, Daphna Michaeli, Assaf Mosquna

**Affiliations:** ^1^The Robert H. Smith Institute of Plant Sciences and Genetics in Agriculture, The Hebrew University of Jerusalem, Rehovot, Israel; ^2^Tel Hai Rodman College, Upper Galilee, Israel

**Keywords:** abscisic acid, pyrabactin resistance 1 (PYR)/PYR1-like (PYL)/regulatory components of ABA receptor (RCAR), plant signaling, PP2C group A, SnRK2, plant evolution

## Abstract

We hereby review the perception and responses to the stress hormone Abscisic acid (ABA), along the trajectory of 500M years of plant evolution, whose understanding may resolve how plants acquired this signaling pathway essential for the colonization of land. ABA levels rise in response to abiotic stresses, coordinating physiological and metabolic responses, helping plants survive stressful environments. In land plants, ABA signaling cascade leads to growth arrest and large-scale changes in transcript levels, required for coping with environmental stressors. This response is regulated by a PYRABACTIN RESISTANCE 1-like (PYL)–PROTEIN PHOSPHATASE 2C (PP2C)–SNF1-RELATED PROTEIN KINASE 2 (SnRK2) module, that initiates phosphor-activation of transcription factors and ion channels. The enzymatic portions of this module (phosphatase and kinase) are functionally conserved from streptophyte algae to angiosperms, whereas the regulatory component –the PYL receptors, putatively evolved in the common ancestor of *Zygnematophyceae* and embryophyte as a constitutive, ABA-independent protein, further evolving into a ligand-activated receptor at the embryophyta. This evolutionary process peaked with the appearance of the strictly ABA-dependent subfamily III stress-triggered angiosperms' dimeric PYL receptors. The emerging picture is that the ancestor of land plants and its predecessors synthesized ABA, as its biosynthetic pathway is conserved between ancestral and current day algae. Despite this ability, it was only the common ancestor of land plants which acquired the hormonal-modulation of PYL activity by ABA. This raises several questions regarding both ABA's function in ABA-non-responsive organisms, and the evolutionary aspects of the ABA signal transduction pathway.

## Introduction

Understanding the evolution of Abscisic acid (ABA) signaling may resolve the puzzle of how plants acquired a major stress signaling pathway that was essential for the colonization of land by ancestral plants. Early land plants, are believed to have been derived from a single common aquatic algal ancestor and had to cope with new challenges, unique to the terrestrial environment (see review by de Vries and Archibald, 2018). Desiccation tolerance was a key adaptive trait for aquatic organisms transitioning to terrestrial environment. This trait has been largely lost in vegetative tissues of trichophytes (see review by Cuming, 2019). Instead, angiosperm adaptation to land was dependent on the ability to regulate the intake and loss of water to the environment (see review by Anderegg, 2015). One major mean by which angiosperms maintain their water balance is the regulation of evapotranspiration *via* the stomata pores. A plant's daily transpiration rhythm is regulated by many internal and external factors, coordinating stomata aperture during its diurnal cycle. The production of the phytohormone ABA in case of an abiotic stress—overwrites transpiration rhythms and results in a rapid closure of the stomata (see review by Brodribb and McAdam, 2017). In the absence of ABA, as in the case of auxotrophic ABA mutants, the ability to close stomata in response to environmental cues is impaired, and as a result, the ability to endure harmful environments is compromised, ultimately deteriorating plant's growth and development (Koornneef et al., 1982; Taylor et al., 1988). Thus, ABA's regulation of land plant water balance is vital for a sessile plant, whose surroundings are in constant change.

The hormone ABA acts through a conserved signal transduction pathway. This pathway is comprised of a PYRABACTIN RESISTANCE 1-Like (PYL)–PROTEIN PHOSPHATASE 2C (PP2C) and SNF1-RELATED PROTEIN KINASE 2 (SnRK2). The binding of ABA to a soluble PYL protein triggers a conformational change that allows the receptors to bind and inhibit the PP2C that normally represses ABA signaling (Melcher et al., 2009; Miyazono et al., 2009; Nishimura et al., 2009; Park et al., 2009; Yin et al., 2009; Melcher et al., 2010; Peterson et al., 2010). This formation of PYL–ABA–PP2C complex, releases SnRK2 from the otherwise inhibitory complex with PP2C, initiating phosphorylation of transcription factors and ion channels, involved in ABA output responses (Fujii et al., 2009; Umezawa et al., 2009; see reviews by Hubbard et al., 2010; Weiner et al., 2010). This review focuses on the physiological and biochemical perception and responses to ABA, along the trajectory of five hundred million years of plant evolution, from streptophyte algae to angiosperms.

## ABA “Output” Response in Algae

The presence of ABA has been confirmed in many chlorophytes and streptophyte algae species, yet the environment-induced synthesis of ABA was demonstrated solely in few aquatic algae species (Tietz and Kasprik, 1986; Hirsch et al., 1989; Hori et al., 2014). ABA biosynthesis in the chlorophytes *Draparnaldia mutabilis* and *Dunahella parua* is induced by salinity, whereas it is seasonally accumulated in the streptophyte algae *Chara contraria* (Sabbatini et al., 1987; Tietz et al., 1989; Hirsch et al., 1989). Despite the evidences of ABA biosynthesis in these aforementioned species, in the vast majority of explored algae, no such significant cellular or physiological function was documented (Hirsch et al., 1989; Tietz et al., 1989; Negin and Moshelion, 2016). This is true even in cases where algae were treated with high dosage of the phytohormone (Kobayashi et al., 1997; Nagao et al., 2008; Sulochana and Arumugam, 2016). Exceptions to note are the alterations of membrane properties of *Nitella* treated with ABA (Wanless et al., 1973; Ord et al., 1977) and the ABA inhibition of *Chara* oospores germination (Sederias and Colman, 2007). Despite these putative adaptive responses to ABA, the effect on growth and other cellular functions in these studies were triggered by the application of high concentrations of ABA ranging from 40 to 500 μM, far from endogenous physiological hormone levels (Ullrich and Kunz, 1984; Saradhi et al., 2000; Yoshida et al., 2003; Yoshida et al., 2004). Thus, these extreme ABA concentrations, required to elicit such responses, are prone to attribute to toxicity rather than a putative activation of an ABA signaling cascade. Taken together, little evidence manifests a clear physiological function for ABA in chlorophyte and streptophyte algae, despite its obvious presence of in both algae phyla.

## ABA “Output” Responses in Early Divergent Land Plants (Bryophytes, Lycophytes, Ferns)

The presence of endogenous ABA, and the response to exogenous ABA application, are well described in bryophytes, but less so in lycophytes and ferns (see reviews by Hartung, 2010; Takezawa et al., 2011; Brodribb and McAdam, 2017). In bryophytes, ectopic ABA application and the genetic mimicking of ABA signaling affects various developmental processes. In liverworts, exogenous ABA inhibits gemma and thalli growth and the establishment and maintenance of gemma dormancy of *Marchantia polymorpha* (Tougane et al., 2010; Eklund et al., 2018). In mosses, ABA elicits protonemal morphological changes. For example, in *Physcomitrella patens*, ABA induces the formation of thick-walled spherical “brood cell”, and it inhibits protonemal differentiation into “leafy” gametophores (Takezawa et al., 2011). The role of ABA in stress tolerance has also been demonstrated in bryophytes. In *M. polymorpha*, the application of ABA improved gemma survival rate following desiccation or freezing, hypothetically resulting from an ABA-induced accumulation of soluble sugars and intracellular rearrangement of vacuoles and chloroplasts (Pence et al., 2005; Hatanaka and Sugawara, 2010; Akter et al., 2014). In addition, the ABA-induced biosynthesis of bisbibenzyls was hypothesized to improve UV irradiation tolerance (Kageyama et al., 2015). Similarly, in a few moss species (*P. patens*, *Funaria hygrometrica*, *Atrichum undulatum* and *Ditrichum cornubicum*) the application of ABA had a positive effect on tolerance to both desiccation and freezing (Takezawa et al., 2011). This adaptation to stress was putatively attributed to the accumulation of protective proteins such as the LATE EMBRYOGENESIS ABUNDANT (LEA), and enzymes associated with osmotic cellular adjustment (Khandelwal et al., 2010; Shinde et al., 2012; Ghosh et al., 2016). Thus, a cellular physiological response to ABA, associated with the adaptation to abiotic stress is evidenced in the first plants habituating land.

The role of ABA in regulating stomata aperture, however, remains ambiguous in earlier divergent plants. Stomata was an “innovation” that facilitated plant terrestrial adaptation. It is generally present in most bryophyte plants except liverworts (see review by Chater et al., 2017). It is thought that the major function of bryophyte stomata was to allow spore capsule desiccation, as the stomata-deficient *PpSMF1* (SPEECHLESS, MUTE and FAMA) mutant of *P. patens* retained water in its sporangia (Chater et al., 2016). Neither ectopic ABA application, nor the darkening of hornworts, triggered their stomata closure. However, hornworts' stomata did respond to reduction in water potential, emphasizing their potent responsiveness to environmental cues (Pressel et al., 2018). The application of 100 µM of exogenous ABA did affect stomata aperture in both *P. patens* and *F. hygrometrica*, however, when ABA signaling was genetically blocked in *P. patens*, neither stomata aperture, nor spore capsule dehydration phenotypes, were reported, suggesting that the ectopically applied hormone levels might not mock on endogenous ABA titrations (Chater et al., 2011; Shinozawa et al., 2019). Taken together, further studies, involving genetic assays, could better clarify the inductive role of ABA in regulating stomata aperture in bryophytes.

In early vascular plants (lycophytes and ferns) there is an active debate regarding the regulation of stomatal aperture by ABA. Stomatal responses to ABA in these plants could be measured only under specific environmental conditions, suggesting a minor contribution of ABA to the regulation of their aperture (Ruszala et al., 2011; Soni et al., 2012; Hõrak et al., 2017). For example, *Selaginella bryopteris* stomata displayed no response to ABA in non-stress conditions, despite its response to ABA under the combination of high temperatures (35 °C) and highly elevated vapor pressure deficit (of 4.5kPa; Soni et al., 2012). A mild reduction in stomata conductance in *Athyrium filix-femina* and *Dryopteris filix-mas* was recorded when treated with 10 μM foliar ABA spraying, however, the authors reported that this response was primarily dependent on cultivation methods (Hõrak et al., 2017). Additional stomatal conductance studies that were correlated with endogenous measurements of ABA levels showed that leaf hydraulics was the predominant factor that primarily regulated stomatal aperture while neither endogenous, nor exogenous ABA, triggered stomata closure in lycophytes and ferns (Brodribb and McAdam, 2011; McAdam and Brodribb, 2012; McAdam et al., 2016; Cardoso et al., 2019). Overall, due to the residual effect of ABA on stomata closure in the aforementioned studies, it seems that in bryophytes and early divergent vascular plants (lycophytes and ferns), stomatal regulation is primarily a hydraulics-driven process.

## The PP2C-SnRK2 Signaling Module Throughout Plant Evolution

The module of the PP2C-SnRK2 phosphatase-kinase is highly conserved throughout plant evolution, preceding the adoption of a regulatory role for the ABA molecule (Tougane et al., 2010; Chater et al., 2011; Hauser et al., 2011; Ruszala et al., 2011; Komatsu et al., 2013; Lind et al., 2015; McAdam et al., 2016; Shinozawa et al., 2019). The cellular signaling of ABA in land plants initiates phosphorylation events mediated by a conserved family of SnRK2 kinases. Vascular plant SnRK2s are classified into three subclasses (Lind et al., 2015; McAdam et al., 2016). Subclass III SnRK2s are pivotal for ABA signaling in *Arabidopsis*, as in the absence of three such family members, there was an absolute shutdown of ABA signaling and response (Fujii and Zhu, 2009). The other SnRK2 subclasses play an important role in osmostress responses in *Arabidopsis* (Fujii et al., 2011). Streptophyte algae and bryophytes encode only subclass III SnRK2s, suggesting that the latter might be the founding members of the family, while the other two subclasses could have been a more recent adaptation of vascular plants (Umezawa et al., 2010; Lind et al., 2015).

An evolutionarily conserved function of subclass III SnRK2 in ABA signaling from streptophyta through angiosperms was demonstrated in multiple genetic studies (Chater et al., 2011; Ruszala et al., 2011; Shinozawa et al., 2019). In the moss *P. patens*, the deletion of *PpSnRK2A/PpOST1* leads to a reduced stomatal response to ectopic ABA, similar to a homologous single *Arabidopsis snrk2.6/ost1* mutant (Chater et al., 2011). *P. patens* quadruple mutant (*snrk2a/b/c/d*) is ABA-insensitive, and it is brood cell development-deficient, lacking both ABA-induced gene expression and desiccation tolerance. Unfortunately, there was no data available regarding the sporophyte ABA response of this mutant, including its stomata response, nor its sporangium dehydration. This strong *P. patens* insensitive phenotype was similar to the *Arabidopsis* triple *snrk2.2/2.3/2.6* mutant (Shinozawa et al., 2019), displaying complete ABA insensitivity. This conserved function of SnRK2 was further exemplified by algae/angiosperm-moss cross-species complementation (Shinozawa et al., 2019). The expression of either of the *Arabidopsis SnRK2.6* gene or the semi-terrestrial alga *Klebsormidium nitens KnOST1* gene complemented *P. patens snrk2* quadruple mutants (Shinozawa et al., 2019). The expression of *PpOST1* from *P. patens* or *SmOST1* from the lycophyte *Selaginella moellendorffii* in *Arabidopsis snrk2.6/ost1* mutant partially rescued stomata ABA insensitivity phenotype (Chater et al., 2011; Ruszala et al., 2011). Taken together, the plant SnRK2s functional conservation likely preceded land habituation.

Furthermore, the SnRK2 phosphorylation targets are also highly conserved throughout plant evolution. The S-type anion channel SLAC1 and ABRE/ABFs transcription factors are SnRK2 substrates in *Arabidopsis* (Furihata et al., 2006; Fujii et al., 2007; Geiger et al., 2009; Lee et al., 2009). SnRK2s from algae (*K. nitens*), liverworts (*M. polymorpha*), moss (*P. patens*), lycophyte (*S. moellendorffii*) and fern (*Ceratopteris richardii*) could activate *Arabidopsis* SLAC1 in *Xenopus laevis* oocytes (Lind et al., 2015; McAdam et al., 2016). However, these SnRK2s cannot activate their native SLACs, suggesting that SnRK2-SLAC1 module for regulating stomata aperture emerged after divergence of ferns and seed plants (Lind et al., 2015; McAdam et al., 2016). SnRK2 ortholog from *K. nitens*, *M. polymorpha* and *P. patens* were capable of transducing ABA-induced gene expression *via* bZIP transcription factor ABF2 in *Arabidopsis* protoplasts (Lind et al., 2015). For detailed ABRE/ABFs evolution see Cuming (2019). In addition, PpSnRK2s from *P. patens* and *Arabidopsis* SnRK2.6/OST1 phosphorylated *in vitro* the same ABA-responsive phosphopeptides (Amagai et al., 2018). Thus, not only is the kinase itself highly conserved, but also the cellular targets of class III SnRK2 are highly conserved, both from algae through angiosperms.

Both the positive and the negative regulatory proteins of the SnRK2 kinase are, too, conserved throughout land plant evolution (Lind et al., 2015; Saruhashi et al., 2015; Yasumura et al., 2015; Stevenson et al., 2016; Lin et al., 2020; Takahashi et al., 2020). Post-translational modifications and protein–protein interactions are the two key regulation means of SnRK2 (Belin et al., 2006; Vlad et al., 2009; Soon et al., 2012; Saruhashi et al., 2015; Stevenson et al., 2016; Nguyen et al., 2019; Lin et al., 2020; Soma et al., 2020; Takahashi et al., 2020). The activation of *Arabidopsis* SnRK2s requires phosphorylation of key serine residues in kinase activation loop (Ser171 and Ser175 for AtSnRK2.6/OST1; Vlad et al., 2010; Soon et al., 2012). In the moss *P. patens*, the activation of SnRK2s is mediated by an ABA Non-Responsive/ABA Responsive Kinase/Constitutive Triple-Response-1-Like (ANR/ARK/CTR1L) protein kinase, a member of B3 Raf-like kinases whose orthologues are conserved in streptophyte algae, but considered lost in vascular plants (Saruhashi et al., 2015; Yasumura et al., 2015; Stevenson et al., 2016; Shinozawa et al., 2019). Recent studies indicated that B2, B3 and B4 groups, are also of the Raf-like kinases family, and are essential for ABA-induced phosphorylation and activation of SnRK2s in *Arabidopsis* (Lin et al., 2020; Takahashi et al., 2020). Thus, this regulation by post-translational modification by Raf-like kinases is too, likely conserved from the common ancestor of algae to land plants.

Functional conservation is also the case for the negative PP2C regulators of SnRK2. In *Arabidopsis*, PP2Cs interacts with SnRK2s and inhibits the kinase activity as it dephosphorylates key serine residues in the kinase activation loop, and physically blocking the kinase catalytic site (Belin et al., 2006; Vlad et al., 2009; Soon et al., 2012). In angiosperms, group A PP2C contains multi-genes with redundant function (Leung et al., 1994; Meyer et al., 1994; Leung et al., 1997; Rodriguez et al., 1998; Saez et al., 2004; Schweighofer et al., 2004; Xue et al., 2008; Zhang et al., 2018; Fujioka et al., 2019). High-order of loss-of-function *Arabidopsis* mutant of PP2C displays an increase of ABA sensitivity, and partially constitutive ABA response (Saez et al., 2006; Rubio et al., 2009). Similarly, in the moss, two group A PP2Cs are encoded by *P. Patens* genome, and the disruption of *PpABI1A* gene results in up-regulation of ABA-induced gene expression and enhanced freezing tolerance (Komatsu et al., 2009). The double mutant *ppabi1a/b* plant shows constitutive “brood cell” phenotype, a global activation of ABA-induced gene expression, and an increase in general protein phosphorylation, indicative of unchecked SnRK2 activity (Komatsu et al., 2013; Amagai et al., 2018). Overexpression of MpABI1 in *M. polymorpha* and *P. patens* resulted in an inhibition of ABA-induced gene expression and reduction of sensitivity of ABA-induced morphological changes (Tougane et al., 2010; Eklund et al., 2018). Moreover, moss and liverwort PP2C phosphatases inhibited SnRK2 activation of *Arabidopsis* SLAC1 expressed in *Xenopus* oocyte (Lind et al., 2015). Albeit all genomes of organisms from the green lineage (Chloroplastida) encode group A PP2Cs (Hauser et al., 2011), little is known about the biochemical interactions of these proteins with SnRK2s in algae. Taken together, these data suggest that PP2C-SnRK2 regulation module is conserved, possibly since the last common ancestor of streptophytes. As aforementioned, since algae do not activate signaling responses to ABA but they do actively transduce downstream signaling components homologous to higher plants', it is likely that the function of SnRK2, its regulatory components, and its cellular targets, preceded that of ABA signaling.

## The Evolution of ABA Receptors in Land Plants

The regulatory unit controlling the aforementioned SnRK2-PP2C module is the most recent evolutionarily among the apex of ABA-signaling-transducing apparatuses. All land plants comprise ABA receptors whose function is largely conserved (Park et al., 2009; Ma et al., 2009; Gonzalez-Guzman et al., 2014; He et al., 2014; Pri-Tal et al., 2017; Mega et al., 2019; Kai et al., 2019). Biochemically, ABA is perceived by a family of Steroidogenic Acute Regulatory Transfer (START)-domain protein receptor called PYRABACTIN RESISTANCE 1/PYR1-LIKE/REGULATORY COMPONENTS OF ABA RECEPTOR (PYR/PYL/RCAR) (Ma et al., 2009; Park et al., 2009). Structure studies reveal a “gate-latch-lock” mechanism that regulate receptor activity: ABA receptors have an open ligand-binding pocket, flanked by two mobile β-loops: the gate and latch. The binding of ABA in the pocket induces a closure of the gate loop and forms a surface that enables the docking of the PP2C co-receptor. A highly conserved tryptophan of PP2C inserts into the “ABA pocket” to further stabilize the PYL-ABA-PP2C ternary complex (Melcher et al., 2009; Miyazono et al., 2009; Yin et al., 2009; Moreno-Alvero et al., 2017). This formation of the ternary complex releases SnRK2 from PP2C inhibition as the PYL and SnRK2 compete on the same PP2C interface (Soon et al., 2012).

In angiosperms, PYL proteins are clustered into three subfamilies, which differ in their affinity to ABA, and in their oligomeric state, each comprised of multiple genes with a partially redundant function. Subfamily III receptor forms a homodimer, whereas subfamily I and II receptors are monomers (Miyazono et al., 2009; Nishimura et al., 2009; Santiago et al., 2009; Szostkiewicz et al., 2010; Hao et al., 2011; He et al., 2014). Mutations resulting in monomer conformation increase the receptor's affinity to ABA and to the PP2C (Dupeux et al., 2011; Hao et al., 2011). Monomeric ABA receptors, in comparison to dimeric receptors, require lower ABA concentration to elicit PP2C inhibition (Okamoto et al., 2013; Gonzalez-Guzman et al., 2014; Pri-Tal et al., 2017; Mega et al., 2019). Based on *in vitro* data, some monomeric receptors have “basal activity”, thereby able to interact and inhibit PP2C activity in the absence of ABA (Hao et al., 2011; Mosquna et al., 2011; Sun et al., 2019). In contrast, dimeric receptors have negligible basal activity, as ABA is required for dimer dissociation (Dupeux et al., 2011; Hao et al., 2011).

Bryophyte receptors are clustered distinctly from vascular plants according to phylogenetic analyses (Weng et al., 2016; Sun et al., 2019). Among vascular plant receptors, subfamily I is phylogenetically closer to that of bryophytes, and subfamily III likely diverged later, as it is unique to angiosperm (Hauser et al., 2011; Weng et al., 2016; Sun et al., 2019). It has been shown that land plant PYL receptors have evolutionarily conserved function (Bowman et al., 2017; Jahan et al., 2019; Shinozawa et al., 2019; Sun et al., 2019). In liverworts, single receptor MpPYL1 knock-out *M. polymorpha* mutant abolished ABA-induced growth inhibition and gene expression (Jahan et al., 2019). The conserved function of MpPYL1 was further confirmed by ABA binding ability, receptor-mediated PP2C inhibition, the activation of ABA-induced gene expression, and the cross-species complementation of *Arabidopsis* ABA-related mutants compromise in either of biosynthetic-pathways or PYL genes (Bowman et al., 2017; Jahan et al., 2019; Sun et al., 2019). Similarly, ABA receptors from the moss *P. Patens* and the lycophyte *S. moellendorffii* displayed both PP2C inhibition and activation of ABA-induced gene expression (Shinozawa et al., 2019; Sun et al., 2019). As the essence of the function of the receptor is the binding to its target, the PP2C interface, the conservation of these targets might themselves have dictated receptor functional conservation, as the SnRK2 and the receptor compete on the very same interface in PP2C.

## The Algal Origin of ABA Receptors

The majority of algae genomes do not encode PYL-like proteins, but few species comprise PYL-like proteins, whose conserved basal, ABA-independent PP2C inhibition activity, suggests that the regulation of PP2C activity might be the ancestral function of the PYL proteins (Sun et al., 2019). Recent genomic and transcriptomic studies demonstrated that some *Zygnematophyceae* algae genome encode PYL homologous proteins (de Vries et al., 2018; Cheng et al., 2019). Protein sequence analysis of these *Zygnematophyceae* PYLs revealed amino acid differences in ABA-binding residues, otherwise conserved in bona fide ABA receptors (Sun et al., 2019). Biochemical and genetic complementation assays, confirmed that *Zygnema circumcarinatum* PYL-like protein (ZcPYL8) can elicit ABA signaling in *Arabidopsis* as it possesses the ability to inhibit PP2C. Further analysis demonstrated that this protein has basal, ABA-independent PP2C inhibition activity, and it could not bind ABA (Sun et al., 2019). Thus, the analysis of the algal PYL indicate that ABA hormonal modulation *via* ligand activation was acquired after the divergence of the ancestor of streptophyte algae from the common ancestor of land plants (Sun et al., 2019).

Studies describe two intertwined trends in the evolution of the ABA receptors: the rise in gene number due to the increase in genetic complexity; and the reduction in receptor ABA-independent basal activity (Sun et al., 2019). We hereby focus on the reduction in receptor basal activity as former reviews by Umezawa et al. (2010) and Hauser et al. (2011) explored the topic of the increase in genetic complexity. The analysis of ABA-independent receptor inhibition of PP2C by PYLs encoded by early divergent plants, suggests a reduction in receptor ABA-independent, constitutive basal activity, in favor of ABA-dependent activity. For example, basal activity of *M. polymorpha* MpPYL1 was around 50% phosphatase inhibition in the absence of ABA. In comparison, three out of four receptors of *S. moellendorffii* had only 15–30% such basal activity, while the fourth SmPYL2 was fully ABA-dependent (Sun et al., 2019). This evolutionary process peaked with the appearance of the strictly ABA-dependent subfamily III dimeric receptors, which are limited to later divergent angiosperms. Lower basal activity provides a broader range of response, and so is the contrary: high basal activity masks the fine-tuned ABA-triggered response. The reduction of ABA-independent basal constitutive activity, alongside the appearance of the dimeric receptors that dominate the response in angiosperms, suggests that a dampening of the basal activity of the receptors was a driving force for the evolution of ABA responsiveness in land plant PYLs (Sun et al., 2019). Thus, in angiosperms, dimeric PYL receptors have evolved, allowing both “finer-tuning” response to variable levels of ABA, and dominating the adaptive stress response of ABA (Park et al., 2009; Okamoto et al., 2013; Pri-Tal et al., 2017; Vaidya et al., 2017; Vaidya et al., 2019).

## Conclusions and Open Questions: The Evolutionary Course of ABA Signaling Module

**Figure 1:**
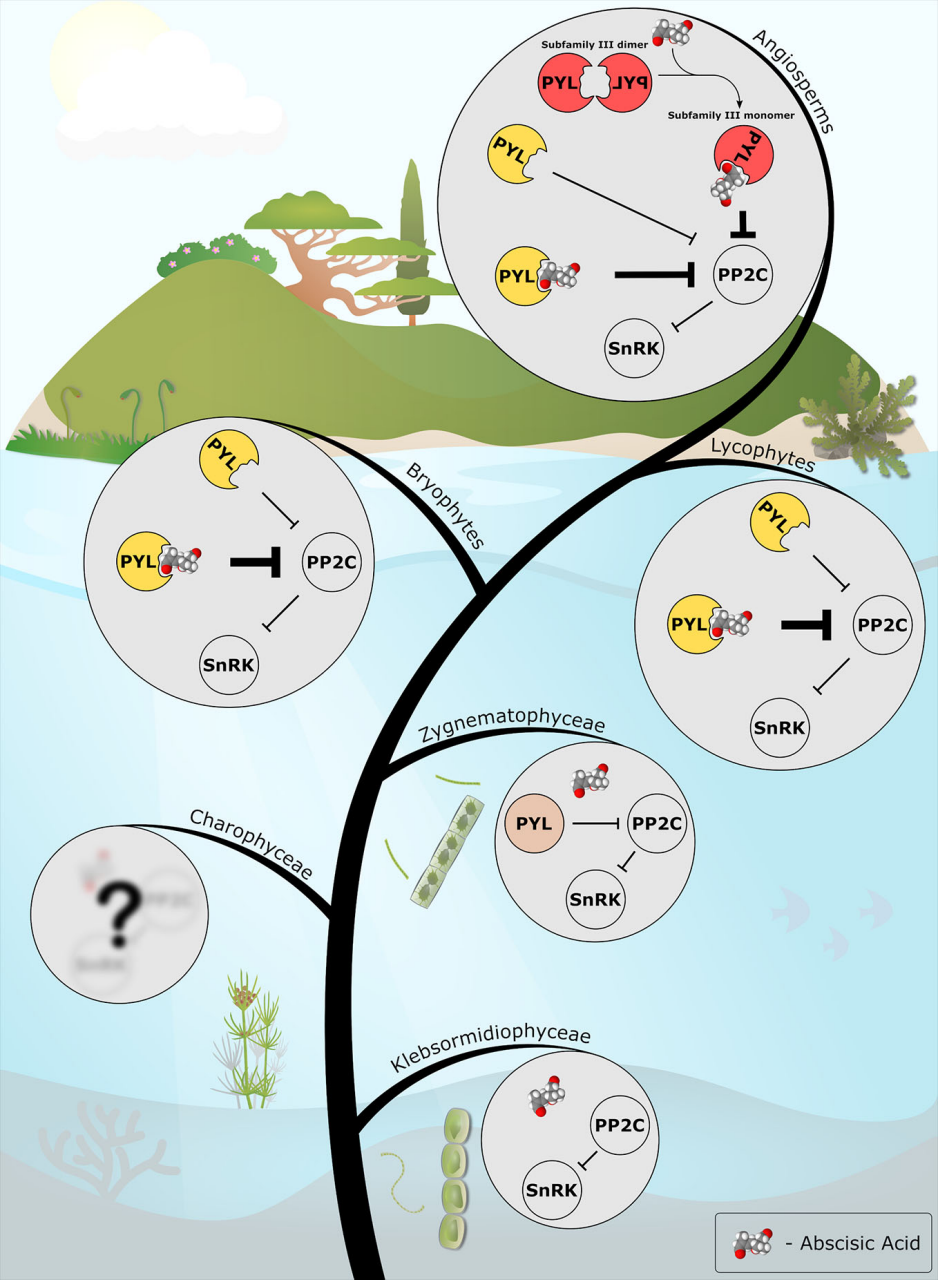
The emerging evolutionary scenario of ABA signaling as described in this review. ABA biosynthesis and PP2C-SnRK2 signaling modules are present in the streptophyte algae (e.g. *Klebsormidiophyceae*). A PYL protein with only basal, ABA-independent, PP2C-inhibition activity (in light brown) evolved in the common ancestor of *Zygnematophyceae* and land plants. Along the course of evolution, the PYL protein of the last common ancestor of land plants (in yellow) gained the ABA-dependent activity, thus recruited ABA into the preexisting signaling cascade. In angiosperms, the appearance of a new subfamily of dimeric PYLs (in red) added another layer of regulation, facilitating ABA-mediated fine-tuning of abiotic stress signaling in plants. ABA molecule is presented as a Van der Waals spheres model. The model was generated with Jmol: an open-source Java viewer for chemical structures in 3D. http://www.jmol.org/.

The collective data from recent years allow us to draw a putative picture of plant ABA signaling evolution (**Figure 1**). It is likely that the common ancestor of *Zygnematophyceae* and embryophytes possessed a PP2C-SnRK2 module that was regulated by a PYL-like protein (also reviewed by Fürst-Jansen et al., 2020). It is still unknown how these organisms regulated the activity of the PYL-like proteins, whether it was by transcriptional, translational or post-translational modifications, or possibly, by allosteric modulation of a yet unidentified hypothetical small molecule. The origin of the PYL proteins is also currently unknown, however, one hypothesis suggests that this ancestral START domain protein was obtained from soil bacteria *via* horizontal gene transfer (Cheng et al., 2019).

The ancestor of land plants and his predecessors synthesized ABA, as its biosynthetic pathway is conserved between ancestral and current algae (Hauser et al., 2011; de Vries et al., 2018). Despite this ability to produce ABA, it was only the common ancestor of land plants whom acquired the hormonally modulation of PYL activity by ABA (Sun et al., 2019). This raises several questions regarding ABA's function in ABA-non-responsive organisms, such as modern day algae, and regarding evolutionary aspects of the ABA signal transduction pathway, such as what made ABA in particular a successful stress transducer?

## Author Contributions

YS, DM, and AM drafted the manuscript. OP-T contributed to the graphics.

## Funding

The Israel Science Foundation (No. 661/18) was the only funding source.

## Conflict of Interest

The authors declare that the research was conducted in the absence of any commercial or financial relationships that could be construed as a potential conflict of interest.

## References

[B1] Akter K. Kato M. Sato Y. Kaneko Y. Takezawa D. (2014). Abscisic acid-induced rearrangement of intracellular structures associated with freezing and desiccation stress tolerance in the liverwort *Marchantia polymorpha*. J. Plant Physiol. 171, 1334–1343. 10.1016/j.jplph.2014.05.004 25046754

[B2] Amagai A. Honda Y. Ishikawa S. Hara Y. Kuwamura M. Shinozawa A. (2018). Phosphoproteomic profiling reveals ABA-responsive phosphosignaling pathways in *Physcomitrella patens*. Plant J. 94, 699–708. 10.1111/tpj.13891 29575231

[B3] Anderegg W. R. (2015). Spatial and temporal variation in plant hydraulic traits and their relevance for climate change impacts on vegetation. New Phytol. 205, 1008–1014. 10.1111/nph.12907 25729797

[B4] Belin C. de Franco P. O. Bourbousse C. Chaignepain S. Schmitter J. M. Vavasseur A. (2006). Identification of features regulating OST1 kinase activity and OST1 function in guard cells. Plant Physiol. 141, 1316–1327. 10.1104/pp.106.079327 16766677 PMC1533939

[B5] Bowman J. L. Kohchi T. Yamato K. T. Jenkins J. Shu S. Ishizaki K. (2017). Insights into land plant evolution garnered from the *Marchantia polymorpha* genome. Cell 171, 287–304 e215. 10.1016/j.cell.2017.09.030 28985561

[B6] Brodribb T. J. McAdam S. A. (2011). Passive origins of stomatal control in vascular plants. Science 331, 582–585. 10.1126/science.1197985 21163966

[B7] Brodribb T. J. McAdam S. A. M. (2017). Evolution of the stomatal regulation of plant water content. Plant Physiol. 174, 639–649. 10.1104/pp.17.00078 28404725 PMC5462025

[B8] Cardoso A. A. Randall J. M. McAdam S. A. (2019). Hydraulics regulate stomatal responses to changes in leaf water status in the fern *Athyrium filix-femina*. Plant Physiol. 179, 533–543. 10.1104/pp.18.01412 30538169 PMC6426430

[B9] Chater C. Kamisugi Y. Movahedi M. Fleming A. Cuming A. C. Gray J. E. (2011). Regulatory mechanism controlling stomatal behavior conserved across 400 million years of land plant evolution. Curr. Biol. 21, 1025–1029. 10.1016/j.cub.2011.04.032 21658944

[B10] Chater C. C. Caine R. S. Tomek M. Wallace S. Kamisugi Y. Cuming A. C. (2016). Origin and function of stomata in the moss *Physcomitrella patens*. Nat. Plants 2, 1–7. 10.1038/nplants.2016.179 PMC5131878 27892923

[B11] Chater C. C. Caine R. S. Fleming A. J. Gray J. E. (2017). Origins and evolution of stomatal development. Plant Physiol. 174, 624–638. 10.1104/pp.17.00183 28356502 PMC5462063

[B12] Cheng S. Xian W. Fu Y. Marin B. Keller J. Wu T. (2019). Genomes of subaerial *Zygnematophyceae* provide insights into land plant evolution. Cell 179, 1057–1067 e1014. 10.1016/j.cell.2019.10.019 31730849

[B13] Cuming A. C. (2019). Evolution of ABA signaling pathways. Abscisic Acid Plants 93, 281–313. 10.1016/bs.abr.2019.06.003

[B14] de Vries J. Archibald J. M. (2018). Plant evolution: landmarks on the path to terrestrial life. New Phytol. 217, 1428–1434. 10.1111/nph.14975 29318635

[B15] de Vries J. Curtis B. A. Gould S. B. Archibald J. M. (2018). Embryophyte stress signaling evolved in the algal progenitors of land plants. Proc. Natl. Acad. Sci. U. S. A. 115, E3471–E3480. 10.1073/pnas.1719230115 29581286 PMC5899452

[B16] Dupeux F. Santiago J. Betz K. Twycross J. Park S. Y. Rodriguez L. (2011). A thermodynamic switch modulates abscisic acid receptor sensitivity. EMBO J. 30, 4171–4184. 10.1038/emboj.2011.294 21847091 PMC3199383

[B17] Eklund D. M. Kanei M. Flores-Sandoval E. Ishizaki K. Nishihama R. Kohchi T. (2018). An evolutionarily conserved abscisic acid signaling pathway regulates dormancy in the liverwort *Marchantia polymorpha*. Curr. Biol. 28, 3691–3699. e3693. 10.1016/j.cub.2018.10.018 30416060

[B18] Fürst-Jansen J. M. de Vries S. Vries J. D. (2020). Evo-physio: on stress responses and the earliest land plants. J. Exp. Bot. 71, 3254–3269. 10.1093/jxb/eraa007 31922568 PMC7289718

[B19] Fujii H. Zhu J. K. (2009). *Arabidopsis* mutant deficient in 3 abscisic acid-activated protein kinases reveals critical roles in growth, reproduction, and stress. Proc. Natl. Acad. Sci. U. S. A. 106, 8380–8385. 10.1073/pnas.0903144106 19420218 PMC2688869

[B20] Fujii H. Verslues P. E. Zhu J.-K. (2007). Identification of two protein kinases required for abscisic acid regulation of seed germination, root growth, and gene expression in *Arabidopsis*. Plant Cell 19, 485–494. 10.1105/tpc.106.048538 17307925 PMC1867333

[B21] Fujii H. Chinnusamy V. Rodrigues A. Rubio S. Antoni R. Park S. Y. (2009). In vitro reconstitution of an abscisic acid signalling pathway. Nature 462, 660–664. 10.1038/nature08599 19924127 PMC2803041

[B22] Fujii H. Verslues P. E. Zhu J. K. (2011). *Arabidopsis* decuple mutant reveals the importance of SnRK2 kinases in osmotic stress responses in vivo. Proc. Natl. Acad. Sci. U. S. A. 108, 1717–1722. 10.1073/pnas.1018367108 21220313 PMC3029766

[B23] Fujioka H. Samejima H. Suzuki H. Mizutani M. Okamoto M. Sugimoto Y. (2019). Aberrant protein phosphatase 2C leads to abscisic acid insensitivity and high transpiration in parasitic *Striga*. Nat. Plants 5, 258–262. 10.1038/s41477-019-0362-7 30804511

[B24] Furihata T. Maruyama K. Fujita Y. Umezawa T. Yoshida R. Shinozaki K. (2006). Abscisic acid-dependent multisite phosphorylation regulates the activity of a transcription activator AREB1. Proc. Natl. Acad. Sci. U. S. A. 103, 1988–1993. 10.1073/pnas.0505667103 16446457 PMC1413621

[B25] Geiger D. Scherzer S. Mumm P. Stange A. Marten I. Bauer H. (2009). Activity of guard cell anion channel SLAC1 is controlled by drought-stress signaling kinase-phosphatase pair. Proc. Natl. Acad. Sci. U. S. A. 106, 21425–21430. 10.1073/pnas.0912021106 19955405 PMC2795561

[B26] Ghosh T. K. Kaneko M. Akter K. Murai S. Komatsu K. Ishizaki K. (2016). Abscisic acid-induced gene expression in the liverwort *Marchantia polymorpha* is mediated by evolutionarily conserved promoter elements. Physiol. Plant. 156, 407–420. 10.1111/ppl.12385 26456006

[B27] Gonzalez-Guzman M. Rodriguez L. Lorenzo-Orts L. Pons C. Sarrion-Perdigones A. Fernandez M. A. (2014). Tomato PYR/PYL/RCAR abscisic acid receptors show high expression in root, differential sensitivity to the abscisic acid agonist quinabactin, and the capability to enhance plant drought resistance. J. Exp. Bot. 65, 4451–4464. 10.1093/jxb/eru219 24863435 PMC4112642

[B28] Hõrak H. Kollist H. Merilo E. (2017). Fern stomatal responses to ABA and CO2 depend on species and growth conditions. Plant Physiol. 174, 672–679. 10.1104/pp.17.00120 28351911 PMC5462029

[B29] Hao Q. Yin P. Li W. Wang L. Yan C. Lin Z. (2011). The molecular basis of ABA-independent inhibition of PP2Cs by a subclass of PYL proteins. Mol. Cell 42, 662–672. 10.1016/j.molcel.2011.05.011 21658606

[B30] Hartung W. (2010). The evolution of abscisic acid (ABA) and ABA function in lower plants, fungi and lichen. Funct. Plant Biol. 37, 806–812. 10.1071/FP10058

[B31] Hatanaka R. Sugawara Y. (2010). Development of desiccation tolerance and vitrification by preculture treatment in suspension-cultured cells of the liverwort *Marchantia polymorpha*. Planta 231, 965–976. 10.1007/s00425-010-1101-5 20101410

[B32] Hauser F. Waadt R. Schroeder J. I. (2011). Evolution of abscisic acid synthesis and signaling mechanisms. Curr. Biol. 21, R346–R355. 10.1016/j.cub.2011.03.015 21549957 PMC3119208

[B33] He Y. Hao Q. Li W. Yan C. Yan N. Yin P. (2014). Identification and characterization of ABA receptors in *Oryza sativa*. PloS One 9, e95246. 10.1371/journal.pone.0095246 24743650 PMC3990689

[B34] Hirsch R. Hartung W. Gimmler H. (1989). Abscisic acid content of algae under stress. Bot. Acta 102, 326–334. 10.1111/j.1438-8677.1989.tb00113.x

[B35] Hori K. Maruyama F. Fujisawa T. Togashi T. Yamamoto N. Seo M. (2014). *Klebsormidium flaccidum* genome reveals primary factors for plant terrestrial adaptation. Nat. Commun. 5, 3978. 10.1038/ncomms4978 24865297 PMC4052687

[B36] Hubbard K. E. Nishimura N. Hitomi K. Getzoff E. D. Schroeder J. I. (2010). Early abscisic acid signal transduction mechanisms: newly discovered components and newly emerging questions. Genes Dev. 24, 1695–1708. 10.1101/gad.1953910 20713515 PMC2922499

[B37] Jahan A. Komatsu K. Wakida-Sekiya M. Hiraide M. Tanaka K. Ohtake R. (2019). Archetypal roles of an abscisic acid receptor in drought and sugar responses in liverworts. Plant Physiol. 179, 317–328. 10.1104/pp.18.00761 30442644 PMC6324230

[B38] Kageyama A. Ishizaki K. Kohchi T. Matsuura H. Takahashi K. (2015). Abscisic acid induces biosynthesis of bisbibenzyls and tolerance to UV-C in the liverwort *Marchantia polymorpha*. Phytochemistry 117, 547–553. 10.1016/j.phytochem.2015.05.009 26055979

[B39] Kai W. Wang J. Liang B. Fu Y. Zheng Y. Zhang W. (2019). PYL9 is involved in the regulation of ABA signaling during tomato fruit ripening. J. Exp. Bot. 70, 6305–6319. 10.1093/jxb/erz396 31504753 PMC6859720

[B40] Khandelwal A. Cho S. H. Marella H. Sakata Y. Perroud P.-F. Pan A. (2010). Role of ABA and ABI3 in desiccation tolerance. Science 327, 546–546. 10.1126/science.1183672 20110497

[B41] Kobayashi M. Hirai N. Kurimura Y. Ohigashi H. Tsuji Y. (1997). Abscisic acid-dependent algal morphogenesis in the unicellular green alga *Haematococcus pluvialis*. Plant Growth Regul. 22, 79–85. 10.1023/A:1005862809711

[B42] Komatsu K. Nishikawa Y. Ohtsuka T. Taji T. Quatrano R. S. Tanaka S. (2009). Functional analyses of the ABI1-related protein phosphatase type 2C reveal evolutionarily conserved regulation of abscisic acid signaling between *Arabidopsis* and the moss *Physcomitrella patens*. Plant Mol. Biol. 70, 327–340. 10.1007/s11103-009-9476-z 19266168

[B43] Komatsu K. Suzuki N. Kuwamura M. Nishikawa Y. Nakatani M. Ohtawa H. (2013). Group A PP2Cs evolved in land plants as key regulators of intrinsic desiccation tolerance. Nat. Commun. 4, 2219. 10.1038/ncomms3219 23900426 PMC3731658

[B44] Koornneef M. Jorna M. Brinkhorst-Van der Swan D. Karssen C. (1982). The isolation of abscisic acid (ABA) deficient mutants by selection of induced revertants in non-germinating gibberellin sensitive lines of *Arabidopsis thaliana* (L.) Heynh. Theor. Appl. Genet. 61, 385–393. 10.1007/BF00272861 24270501

[B45] Lee S. C. Lan W. Buchanan B. B. Luan S. (2009). A protein kinase-phosphatase pair interacts with an ion channel to regulate ABA signaling in plant guard cells. Proc. Natl. Acad. Sci. U. S. A. 106, 21419–21424. 10.1073/pnas.0910601106 19955427 PMC2795491

[B46] Leung J. Bouvier-Durand M. Morris P. C. Guerrier D. Chefdor F. Giraudat J. (1994). *Arabidopsis* ABA response gene ABI1: features of a calcium-modulated protein phosphatase. Science 264, 1448–1452. 10.1126/science.7910981 7910981

[B47] Leung J. Merlot S. Giraudat J. (1997). The *Arabidopsis* ABSCISIC ACID-INSENSITIVE2 (ABI2) and ABI1 genes encode homologous protein phosphatases 2C involved in abscisic acid signal transduction. Plant Cell 9, 759–771. 10.1105/tpc.9.5.759 9165752 PMC156954

[B48] Lin Z. Li Y. Zhang Z. Liu X. Hsu C. C. Du Y. (2020). A RAF-SnRK2 kinase cascade mediates early osmotic stress signaling in higher plants. Nat. Commun. 11, 613. 10.1038/s41467-020-14477-9 32001690 PMC6992735

[B49] Lind C. Dreyer I. López-Sanjurjo E. J. von Meyer K. Ishizaki K. Kohchi T. (2015). Stomatal guard cells co-opted an ancient ABA-dependent desiccation survival system to regulate stomatal closure. Curr. Biol. 25, 928–935. 10.1016/j.cub.2015.01.067 25802151

[B50] Ma Y. Szostkiewicz I. Korte A. Moes D. Yang Y. Christmann A. (2009). Regulators of PP2C phosphatase activity function as abscisic acid sensors. Science 324, 1064–1068. 10.1126/science.1172408 19407143

[B51] McAdam S. A. Brodribb T. J. (2012). Fern and lycophyte guard cells do not respond to endogenous abscisic acid. Plant Cell 24, 1510–1521. 10.1105/tpc.112.096404 22517320 PMC3398560

[B52] McAdam S. A. Brodribb T. J. Banks J. A. Hedrich R. Atallah N. M. Cai C. (2016). Abscisic acid controlled sex before transpiration in vascular plants. Proc. Natl. Acad. Sci. U. S. A. 113, 12862–12867. 10.1073/pnas.1606614113 27791082 PMC5111647

[B53] Mega R. Abe F. Kim J. S. Tsuboi Y. Tanaka K. Kobayashi H. (2019). Tuning water-use efficiency and drought tolerance in wheat using abscisic acid receptors. Nat. Plants 5, 153–159. 10.1038/s41477-019-0361-8 30737511

[B54] Melcher K. Ng L. M. Zhou X. E. Soon F. F. Xu Y. Suino-Powell K. M. (2009). A gate-latch-lock mechanism for hormone signalling by abscisic acid receptors. Nature 462, 602–608. 10.1038/nature08613 19898420 PMC2810868

[B55] Melcher K. Xu Y. Ng L. M. Zhou X. E. Soon F. F. Chinnusamy V. (2010). Identification and mechanism of ABA receptor antagonism. Nat. Struct. Mol. Biol. 17, 1102–1108. 10.1038/nsmb.1887 20729862 PMC2933329

[B56] Meyer K. Leube M. P. Grill E. (1994). A protein phosphatase 2C involved in ABA signal transduction in *Arabidopsis thaliana*. Science 264, 1452–1455. 10.1126/science.8197457 8197457

[B57] Miyazono K. Miyakawa T. Sawano Y. Kubota K. Kang H. J. Asano A. (2009). Structural basis of abscisic acid signalling. Nature 462, 609–614. 10.1038/nature08583 19855379

[B58] Moreno-Alvero M. Yunta C. Gonzalez-Guzman M. Lozano-Juste J. Benavente L. J. Arbona V. (2017). Structure of ligand-bound intermediates of crop ABA receptors highlights PP2C as necessary ABA co-receptor. Mol. Plant 10, 1250–1253. 10.1016/j.molp.2017.07.004 28736053

[B59] Mosquna A. Peterson F. C. Park S. Y. Lozano-Juste J. Volkman B. F. Cutler S. R. (2011). Potent and selective activation of abscisic acid receptors in vivo by mutational stabilization of their agonist-bound conformation. Proc. Natl. Acad. Sci. U. S. A. 108, 20838–20843. 10.1073/pnas.1112838108 22139369 PMC3251050

[B60] Nagao M. Matsui K. Uemura M. (2008). Klebsormidium flaccidum, a charophycean green alga, exhibits cold acclimation that is closely associated with compatible solute accumulation and ultrastructural changes. Plant Cell Environ. 31, 872–885. 10.1111/j.1365-3040.2008.01804.x 18315534

[B61] Negin B. Moshelion M. (2016). The evolution of the role of ABA in the regulation of water-use efficiency: From biochemical mechanisms to stomatal conductance. Plant Sci. 251, 82–89. 10.1016/j.plantsci.2016.05.007 27593466

[B62] Nguyen Q. T. C. Lee S.-J. Choi S.-W. Na Y.-J. Song M.-R. Hoang Q. T. N. (2019). *Arabidopsis* Raf-like kinase raf10 is a regulatory component of core ABA signaling. Mol. Cells 42, 646–660. 10.14348/molcells.2019.0173 31480825 PMC6776158

[B63] Nishimura N. Hitomi K. Arvai A. S. Rambo R. P. Hitomi C. Cutler S. R. (2009). Structural mechanism of abscisic acid binding and signaling by dimeric PYR1. Science 326, 1373–1379. 10.1126/science.1181829 19933100 PMC2835493

[B64] Okamoto M. Peterson F. C. Defries A. Park S. Y. Endo A. Nambara E. (2013). Activation of dimeric ABA receptors elicits guard cell closure, ABA-regulated gene expression, and drought tolerance. Proc. Natl. Acad. Sci. U. S. A. 110, 12132–12137. 10.1073/pnas.1305919110 23818638 PMC3718107

[B65] Ord G. S. G. Cameron I. Fensom D. (1977). The effect of pH and ABA on the hydraulic conductivity of *Nitella* membranes. Can. J. Bot. 55, 1–4. 10.1139/b77-001

[B66] Park S. Y. Fung P. Nishimura N. Jensen D. R. Fujii H. Zhao Y. (2009). Abscisic acid inhibits type 2C protein phosphatases via the PYR/PYL family of START proteins. Science 324, 1068–1071. 10.1126/science.1173041 19407142 PMC2827199

[B67] Pence V. C. Dunford S. S. Redella S. (2005). Differential effects of abscisic acid on desiccation tolerance and carbohydrates in three species of liverworts. J. Plant Physiol. 162, 1331–1337. 10.1016/j.jplph.2005.02.002 16425451

[B68] Peterson F. C. Burgie E. S. Park S. Y. Jensen D. R. Weiner J. J. Bingman C. A. (2010). Structural basis for selective activation of ABA receptors. Nat. Struct. Mol. Biol. 17, 1109–1113. 10.1038/nsmb.1898 20729860 PMC2933299

[B69] Pressel S. Renzaglia K. S. Clymo R. S. Duckett J. G. (2018). Hornwort stomata do not respond actively to exogenous and environmental cues. Ann. Bot. 122, 45–57. 10.1093/aob/mcy045 29897395 PMC6025193

[B70] Pri-Tal O. Shaar-Moshe L. Wiseglass G. Peleg Z. Mosquna A. (2017). Non-redundant functions of the dimeric ABA receptor BdPYL1 in the grass *Brachypodium*. Plant J. 92, 774–786. 10.1111/tpj.13714 28891214

[B71] Rodriguez P. L. Leube M. P. Grill E. (1998). Molecular cloning in *Arabidopsis thaliana* of a new protein phosphatase 2C (PP2C) with homology to ABI1 and ABI2. Plant Mol. Biol. 38, 879–883. 10.1023/A:1006012218704 9862504

[B72] Rubio S. Rodrigues A. Saez A. Dizon M. B. Galle A. Kim T. H. (2009). Triple loss of function of protein phosphatases type 2C leads to partial constitutive response to endogenous abscisic acid. Plant Physiol. 150, 1345–1355. 10.1104/pp.109.137174 19458118 PMC2705020

[B73] Ruszala E. M. Beerling D. J. Franks P. J. Chater C. Casson S. A. Gray J. E. (2011). Land plants acquired active stomatal control early in their evolutionary history. Curr. Biol. 21, 1030–1035. 10.1016/j.cub.2011.04.044 21658945

[B74] Sabbatini M. Argüello J. Fernandez O. Bottini R. (1987). Dormancy and growth-inhibitor levels in oospores of *Chara contraria* A. Braun ex Kütz.(Charophyta). Aquat. Bot. 28 (2), 189–194. 10.1016/0304-3770(87)90040-4

[B75] Saez A. Apostolova N. Gonzalez-Guzman M. Gonzalez-Garcia M. P. Nicolas C. Lorenzo O. (2004). Gain-of-function and loss-of-function phenotypes of the protein phosphatase 2C HAB1 reveal its role as a negative regulator of abscisic acid signaling. Plant J. 37, 354–369. 10.1046/j.1365-313X.2003.01966.x 14731256

[B76] Saez A. Robert N. Maktabi M. H. Schroeder J. I. Serrano R. Rodriguez P. L. (2006). Enhancement of abscisic acid sensitivity and reduction of water consumption in *Arabidopsis* by combined inactivation of the protein phosphatases type 2C ABI1 and HAB1. Plant Physiol. 141, 1389–1399. 10.1104/pp.106.081018 16798945 PMC1533955

[B77] Santiago J. Dupeux F. Round A. Antoni R. Park S. Y. Jamin M. (2009). The abscisic acid receptor PYR1 in complex with abscisic acid. Nature 462, 665–668. 10.1038/nature08591 19898494

[B78] Saradhi P. P. Suzuki I. Katoh A. Sakamoto A. Sharmila P. Shi D. J. (2000). Protection against the photo-induced inactivation of the photosystem II complex by abscisic acid. Plant Cell Environ. 23, 711–718. 10.1046/j.1365-3040.2000.00579.x

[B79] Saruhashi M. Ghosh T. K. Arai K. Ishizaki Y. Hagiwara K. Komatsu K. (2015). Plant Raf-like kinase integrates abscisic acid and hyperosmotic stress signaling upstream of SNF1-related protein kinase2. Proc. Natl. Acad. Sci. U. S. A. 112, E6388–E6396. 10.1073/pnas.1511238112 26540727 PMC4655548

[B80] Schweighofer A. Hirt H. Meskiene I. (2004). Plant PP2C phosphatases: emerging functions in stress signaling. Trends Plant Sci. 9, 236–243. 10.1016/j.tplants.2004.03.007 15130549

[B81] Sederias J. Colman B. (2007). The interaction of light and low temperature on breaking the dormancy of *Chara vulgaris* oospores. Aquat. Bot. 87, 229–234. 10.1016/j.aquabot.2007.06.008

[B82] Shinde S. Nurul Islam M. Ng C. K. Y. (2012). Dehydration stress-induced oscillations in LEA protein transcripts involves abscisic acid in the moss, *Physcomitrella patens*. New Phytol. 195, 321–328. 10.1111/j.1469-8137.2012.04193.x 22591374

[B83] Shinozawa A. Otake R. Takezawa D. Umezawa T. Komatsu K. Tanaka K. (2019). SnRK2 protein kinases represent an ancient system in plants for adaptation to a terrestrial environment. Commun. Biol. 2, 1–13. 10.1038/s42003-019-0281-1 30675528 PMC6340887

[B84] Soma F. Takahashi F. Suzuki T. Shinozaki K. Yamaguchi-Shinozaki K. (2020). Plant Raf-like kinases regulate the mRNA population upstream of ABA-unresponsive SnRK2 kinases under drought stress. Nat. Commun. 11, 1–12. 10.1038/s41467-020-15239-3 32170072 PMC7069986

[B85] Soni D. K. Ranjan S. Singh R. Khare P. B. Pathre U. V. Shirke P. A. (2012). Photosynthetic characteristics and the response of stomata to environmental determinants and ABA in *Selaginella bryopteris*, a resurrection spike moss species. Plant Sci. 191, 43–52. 10.1016/j.plantsci.2012.04.011 22682564

[B86] Soon F. F. Ng L. M. Zhou X. E. West G. M. Kovach A. Tan M. H. (2012). Molecular mimicry regulates ABA signaling by SnRK2 kinases and PP2C phosphatases. Science 335, 85–88. 10.1126/science.1215106 22116026 PMC3584687

[B87] Stevenson S. R. Kamisugi Y. Trinh C. H. Schmutz J. Jenkins J. W. Grimwood J. (2016). Genetic analysis of *Physcomitrella patens* identifies ABSCISIC ACID NON-RESPONSIVE, a regulator of aba responses unique to basal land plants and required for desiccation tolerance. Plant Cell 28, 1310–1327. 10.1105/tpc.16.00091 27194706 PMC4944411

[B88] Sulochana S. B. Arumugam M. (2016). Influence of abscisic acid on growth, biomass and lipid yield of *Scenedesmus quadricauda* under nitrogen starved condition. Biores. Technol. 213, 198–203. 10.1016/j.biortech.2016.02.078 26949054

[B89] Sun Y. Harpazi B. Wijerathna-Yapa A. Merilo E. de Vries J. Michaeli D. (2019). A ligand-independent origin of abscisic acid perception. Proc. Natl. Acad. Sci. U. S. A. 116, 24892–24899. 10.1073/pnas.1914480116 31744875 PMC6900503

[B90] Szostkiewicz I. Richter K. Kepka M. Demmel S. Ma Y. Korte A. (2010). Closely related receptor complexes differ in their ABA selectivity and sensitivity. Plant J. 61, 25–35. 10.1111/j.1365-313X.2009.04025.x 19769575

[B91] Takahashi Y. Zhang J. Hsu P. K. Ceciliato P. H. O. Zhang L. Dubeaux G. (2020). MAP3Kinase-dependent SnRK2-kinase activation is required for abscisic acid signal transduction and rapid osmotic stress response. Nat. Commun. 11, 12. 10.1038/s41467-019-13875-y 31896774 PMC6940395

[B92] Takezawa D. Komatsu K. Sakata Y. (2011). ABA in bryophytes: how a universal growth regulator in life became a plant hormone? J. Plant Res. 124, 437–453. 10.1007/s10265-011-0410-5 21416316

[B93] Taylor I. Linforth R. Al-Naieb R. Bowman W. Marples B. (1988). The wilty tomato mutants *flacca* and *sitiens* are impaired in the oxidation of ABA-aldehyde to ABA. Plant Cell Environ. 11, 739–745. 10.1111/j.1365-3040.1988.tb01158.x

[B94] Tietz A. Kasprik W. (1986). Identification of abscisic acid in a green alga. Biochem. und Physiol. der Pflanzen 181, 269–274. 10.1016/S0015-3796(86)80093-2

[B95] Tietz A. Ruttkowski U. Kohler R. Kasprik W. (1989). Further investigations on the occurrence and the effects of abscisic acid in algae. Biochem. und Physiol. der Pflanzen 184, 259–266. 10.1016/S0015-3796(89)80011-3

[B96] Tougane K. Komatsu K. Bhyan S. B. Sakata Y. Ishizaki K. Yamato K. T. (2010). Evolutionarily conserved regulatory mechanisms of abscisic acid signaling in land plants: characterization of ABSCISIC ACID INSENSITIVE1-like type 2C protein phosphatase in the liverwort *Marchantia polymorpha*. Plant Physiol. 152, 1529–1543. 10.1104/pp.110.153387 20097789 PMC2832234

[B97] Ullrich W. R. Kunz G. (1984). Effect of abscisic acid on nitrate uptake, respiration and photosynthesis in green algae. Plant Sci. Lett. 37, 9–14. 10.1016/0304-4211(84)90195-0

[B98] Umezawa T. Sugiyama N. Mizoguchi M. Hayashi S. Myouga F. Yamaguchi-Shinozaki K. (2009). Type 2C protein phosphatases directly regulate abscisic acid-activated protein kinases in *Arabidopsis*. Proc. Natl. Acad. Sci. U. S. A. 106, 17588–17593. 10.1073/pnas.0907095106 19805022 PMC2754379

[B99] Umezawa T. Nakashima K. Miyakawa T. Kuromori T. Tanokura M. Shinozaki K. (2010). Molecular basis of the core regulatory network in ABA responses: sensing, signaling and transport. Plant Cell Physiol. 51, 1821–1839. 10.1093/pcp/pcq156 20980270 PMC2978318

[B100] Vaidya A. S. Peterson F. C. Yarmolinsky D. Merilo E. Verstraeten I. Park S.-Y. (2017). A rationally designed agonist defines subfamily IIIA abscisic acid receptors as critical targets for manipulating transpiration. ACS Chem. Biol. 12, 2842–2848. 10.1021/acschembio.7b00650 28949512

[B101] Vaidya A. S. Helander J. D. Peterson F. C. Elzinga D. Dejonghe W. Kaundal A. (2019). Dynamic control of plant water use using designed ABA receptor agonists. Science 366, eaaw8848. 10.1126/science.aaw8848 31649167

[B102] Vlad F. Rubio S. Rodrigues A. Sirichandra C. Belin C. Robert N. (2009). Protein phosphatases 2C regulate the activation of the Snf1-related kinase OST1 by abscisic acid in *Arabidopsis*. Plant Cell 21, 3170–3184. 10.1105/tpc.109.069179 19855047 PMC2782292

[B103] Vlad F. Droillard M. J. Valot B. Khafif M. Rodrigues A. Brault M. (2010). Phospho-site mapping, genetic and in planta activation studies reveal key aspects of the different phosphorylation mechanisms involved in activation of SnRK2s. Plant J. 63, 778–790. 10.1111/j.1365-313X.2010.04281.x 20561261

[B104] Wanless I. Bryniak N. Fensom D. (1973). The effect of some growth-regulating compounds upon electroosmotic measurements, transcellular water flow, and Na, K, and Cl influx in *Nitella flexilis*. Can. J. Bot. 51, 1055–1070. 10.1139/b73-130

[B105] Weiner J. J. Peterson F. C. Volkman B. F. Cutler S. R. (2010). Structural and functional insights into core ABA signaling. Curr. Opin. Plant Biol. 13, 495–502. 10.1016/j.pbi.2010.09.007 20934900 PMC2971662

[B106] Weng J. K. Ye M. Li B. Noel J. P. (2016). Co-evolution of hormone metabolism and signaling networks expands plant adaptive plasticity. Cell 166, 881–893. 10.1016/j.cell.2016.06.027 27518563

[B107] Xue T. Wang D. Zhang S. Ehlting J. Ni F. Jakab S. (2008). Genome-wide and expression analysis of protein phosphatase 2C in rice and *Arabidopsis*. BMC Genomics 9, 550. 10.1186/1471-2164-9-550 19021904 PMC2612031

[B108] Yasumura Y. Pierik R. Kelly S. Sakuta M. Voesenek L. A. Harberd N. P. (2015). An ancestral role for CONSTITUTIVE TRIPLE RESPONSE1 proteins in both ethylene and abscisic acid signaling. Plant Physiol. 169, 283–298. 10.1104/pp.15.00233 26243614 PMC4577374

[B109] Yin P. Fan H. Hao Q. Yuan X. Wu D. Pang Y. (2009). Structural insights into the mechanism of abscisic acid signaling by PYL proteins. Nat. Struct. Mol. Biol. 16, 1230–1236. 10.1038/nsmb.1730 19893533

[B110] Yoshida K. Igarashi E. Mukai M. Hirata K. Miyamoto K. (2003). Induction of tolerance to oxidative stress in the green alga, *Chlamydomonas reinhardtii*, by abscisic acid. Plant Cell Environ. 26, 451–457. 10.1046/j.1365-3040.2003.00976.x

[B111] Yoshida K. Igarashi E. Wakatsuki E. Miyamoto K. Hirata K. (2004). Mitigation of osmotic and salt stresses by abscisic acid through reduction of stress-derived oxidative damage in *Chlamydomonas reinhardtii*. Plant Sci. 167, 1335–1341. 10.1016/j.plantsci.2004.07.002

[B112] Zhang Y. Li Q. Jiang L. Kai W. Liang B. Wang J. (2018). Suppressing type 2C protein phosphatases alters fruit ripening and the stress response in tomato. Plant Cell Physiol. 59, 142–154. 10.1093/pcp/pcx169 29121241

